# Fresh Genetic Insights Into Micronutrients Influences on Restless Legs Syndrome Risk

**DOI:** 10.1002/fsn3.70568

**Published:** 2025-08-30

**Authors:** Yun Lin, Haohao Chen, Xiaorui Cai, Xiaoling Tang

**Affiliations:** ^1^ Department of Pharmacy Shantou Central Hospital Shantou Guangdong Province China; ^2^ Department of Pharmacy The First Affiliated Hospital of Shantou University Medical College Shantou Guangdong Province China; ^3^ Department of Pharmacology Shantou University Medical College Shantou Guangdong Province China; ^4^ Department of Pharmacy The Affiliated Cancer Hospital of Shantou University Medical College Shantou Guangdong Province China; ^5^ Department of Nephrology Shantou Central Hospital Shantou Guangdong Province China

**Keywords:** folate, genome‐wide association studies, magnesium, Mendelian randomization, micronutrients, restless legs syndrome, retinol

## Abstract

Restless legs syndrome (RLS) has a multifactorial etiology, and current treatments are suboptimal. Micronutrients influence neuromuscular and dopaminergic function, yet their causal role in RLS is uncertain. This study aimed to investigate whether circulating micronutrients causally influence the risk of RLS by applying an integrated two‐sample, bidirectional, and multivariable Mendelian Randomization (MR) strategy. Genetic instruments for micronutrient levels were obtained from genome‐wide association studies (GWAS) in the IEU Open GWAS database. RLS outcome data were from the EU‐RLS‐GENE consortium (7248 cases, 19,802 controls of European ancestry). Single nucleotide polymorphisms significantly associated with each micronutrient (*p* < 5 × 10^−6^) served as instrumental variables. Primary MR analyses employed the inverse‐variance weighted (IVW) method, supplemented by the robust adjusted profile score (RAPS), MR‐PRESSO, weighted median, MR‐Egger regression, and IVW radial analyses. Bidirectional and multivariable MR were also performed. Sensitivity analyses for heterogeneity and horizontal pleiotropy were conducted to ensure robustness. No micronutrient passed FDR < 0.05, but three achieved suggestive evidence. Higher genetically predicted retinol and magnesium were associated with lower RLS risk (retinol OR 0.46, 95% CI 0.25–0.86; magnesium OR 0.62, 0.39–0.98). After excluding two pleiotropic SNPs, higher folate was associated with higher risk (OR 1.48, 1.10–2.00). Reverse MR showed no effect of RLS liability on these nutrients. In multivariable MR, folate remained positively associated (OR 2.88, 1.07–7.77) and magnesium inversely associated (OR 0.38, 0.15–0.98); the retinol signal weakened (OR 0.55, 0.30–1.01). No other micronutrient demonstrated a causal link. Sensitivity tests showed no material heterogeneity or directional pleiotropy. Genetic evidence supports folate excess and magnesium insufficiency as independent, potentially modifiable contributors to RLS, whereas any protective effect of retinol appears sensitive to joint modeling. These findings warrant replication in non‐European cohorts and mechanistic studies addressing brain‐specific micronutrient regulation.

## Graphical abstract

Applying an integrated Mendelian Randomization approach, we investigated the causal influence of circulating micronutrients on restless legs syndrome (RLS) risk. Our genetic evidence suggests that higher folate increases RLS risk and higher magnesium is protective, whereas retinol's effect was less robust in multivariable models. These findings pinpoint specific micronutrients warranting further research for RLS prevention or treatment.

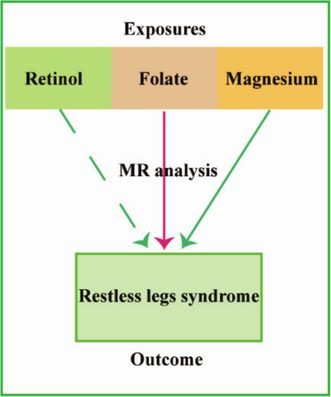

## 1 Introduction

Restless legs syndrome (RLS) is a neurological condition involving both sensory and motor symptoms, where individuals experience a compelling urge to move their legs accompanied by uncomfortable sensations such as tingling, burning, or creeping feelings beneath the skin (Allen et al. 2014; Manconi et al. 2021). These symptoms typically become more severe when the individual is inactive—such as during sitting or lying down—especially in the evening or at night, and are partially or temporarily relieved by movement. RLS affects approximately 4%–29% of the adult population in Western countries, significantly impacting sleep quality, daily functioning, and overall quality of life (AlShareef 2022; Ohayon et al. 2012).

Although the pathophysiology of RLS remains incompletely understood, it appears to involve intricate interactions among genetic predispositions, neurological pathways, and environmental influences (Didato et al. 2020; Mogavero et al. 2024; Tan et al. 2024). Dopaminergic dysfunction and iron deficiency in the central nervous system are among the most studied mechanisms (Earley et al. 2022; Porras and Rouault 2022). However, current treatments focusing on dopaminergic agents and iron supplementation do not fully address the condition; some patients experience inadequate relief or adverse side effects (Gossard et al. 2021; Lv et al. 2021; Makharia et al. 2024; Vlasie et al. 2022). This underscores the need to explore additional factors that may contribute to RLS development and progression.

Micronutrients, including vitamins and minerals, play crucial roles in neuromuscular function, neurotransmitter synthesis, and neuronal health (Bruins et al. 2019; Ji et al. 2017; Makharia et al. 2024; Quan et al. 2023). Deficiencies or imbalances in these nutrients can lead to neurological disorders and exacerbate existing conditions (Huskisson et al. 2007; Levi et al. 2024; Tsalamandris et al. 2023). For instance, iron is essential for dopamine synthesis, and a lack of iron has been associated with the manifestation of RLS symptoms (Connor et al. 2017). Magnesium, for example, is vital for muscle relaxation and nerve impulse transmission, modulating neurotransmitter balance, which is highly relevant to RLS pathophysiology (Fatima et al. 2024; Kirkland et al. 2018). Although it has a plausible physiological role, a systematic review found mixed results from clinical trials, making its overall effectiveness for RLS unclear (González‐Parejo et al. 2024; Marshall et al. 2019). Indeed, observational studies concerning various other micronutrients have often yielded similarly inconsistent findings regarding their role in RLS.

Mendelian Randomization (MR) offers a robust method to evaluate causal effects by employing genetic variants as proxies for exposures of interest (Smith and Ebrahim 2003). This method reduces biases from confounding factors and reverses causation common in observational research, providing more reliable evidence on the effects of exposures like micronutrient levels on disease outcomes (Boehm and Zhou 2022). We utilized an integrated two‐sample, bidirectional, and multivariable MR (MVMR) strategy to explore possible causal links between serum concentrations of eight vitamins—including retinol, carotene, B6, B12, C, D, E, and folate—as well as seven minerals such as calcium, iron, magnesium, potassium, selenium, zinc, and copper—and the risk of developing RLS. By harnessing summary statistics from genome‐wide association studies (GWAS), we aimed to elucidate the role of these micronutrients in RLS pathophysiology, potentially identifying avenues for targeted nutritional interventions.

## 2 Methods

### 2.1 Study Overview

**FIGURE 1:**
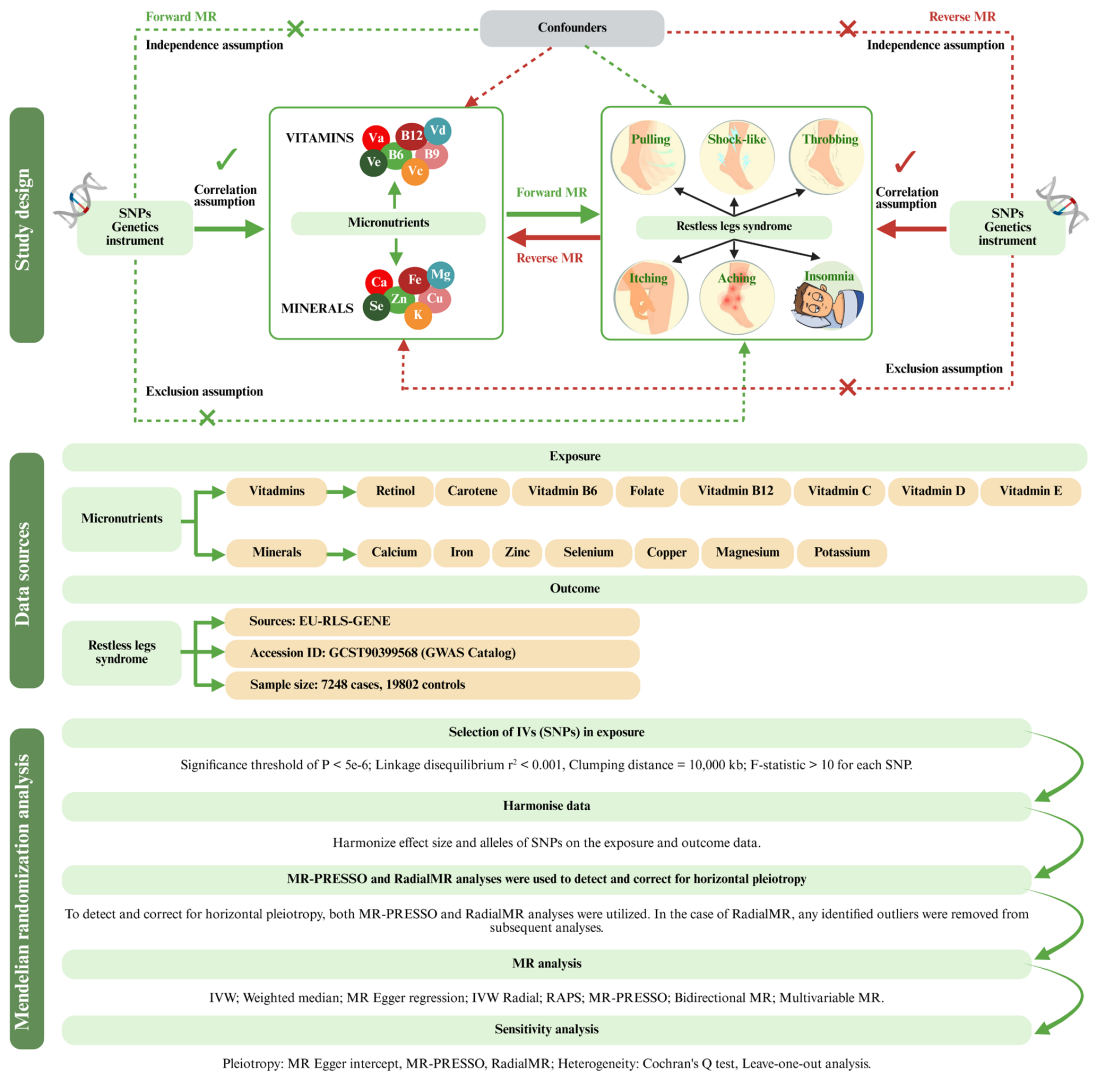
Overview of the methodological framework, data utilized, and analytical workflow for the MR study investigating the causal relationship between micronutrient levels and restless legs syndrome (RLS). The methodological framework illustrates the key conditions required for SNPs to serve as appropriate IVs: they must be associated with the exposure trait, be independent of confounding variables, and influence the outcome trait solely through the exposure pathway. The data utilized section details the specific micronutrients examined, encompassing various vitamins and mineral ions, along with information on the RLS outcome dataset. The analytical workflow outlines the sequential steps undertaken, starting from SNP selection and data harmonization, followed by assessments for pleiotropy and heterogeneity to identify and address potential biases. This is followed by the application of primary MR analytical methods and concluded with sensitivity analyses to evaluate the validity and robustness of the causal relationships identified. GWAS, genome‐wide association study; IVs, instrumental variables; IVW, inverse variance weighted; MR, mendelian randomization; MR‐PRESSO, mendelian randomization pleiotropy residual sum and outlier; RAPS, robust adjusted profile score; SNP, single‐nucleotide polymorphism.

In this study, we investigated whether circulating micronutrients causally influence the risk of RLS by applying an integrated two‐sample, bidirectional, and multivariable MR strategy. Specifically, we examined eight vitamins and seven mineral ions as potential risk factors for RLS, given their critical roles in neuromuscular function, which is central to the pathophysiology of RLS. Our MR analysis is based on three core assumptions as outlined in previous studies (Richmond and Davey Smith 2022; Skrivankova et al. 2021). To enhance the robustness and reliability of our findings, we perform comprehensive sensitivity assessments. These analyses are designed to assess and address potential issues related to horizontal pleiotropy and heterogeneity among the genetic instruments. By applying multiple MR methods and statistical tests, we aim to validate the consistency of our results and mitigate the influence of any biases. Figure 1 provides a detailed visual representation of our study framework and analytical approach.

### 2.2 Data Sources

We analyzed 15 serum micronutrients: eight vitamins—retinol, carotene, vitamins B6, B12, C, D, E, and folate—and seven minerals—calcium, iron, zinc, selenium, copper, magnesium, and potassium. GWAS summary statistics for each micronutrient were obtained from the IEU Open GWAS repository (https://gwas.mrcieu.ac.uk/). The outcome dataset was the discovery meta‐analysis of RLS reported by the EU‐RLS‐GENE consortium and is available under NHGRI‐EBI accession GCST90399568 (Schormair et al. 2024). It includes 7248 clinically diagnosed RLS cases (34.2% male) and 19,802 controls (52.6% male) of European ancestry, recruited in multiple European countries, Québec (Canada), and the United States. Diagnoses were made by neurologists or sleep specialists using the International Restless Legs Syndrome Study Group criteria. A summary of each dataset is provided in Table 1; full trait definitions and covariate adjustments are described in the original publications.

**TABLE 1:** Detailed characteristics of GWAS datasets used in Mendelian randomization analysis.

GWAS ID	Trait	First author	Sex	PMID	Population	Sample size	Year	SNPs
ukb‐b‐17406	Retinol	Ben Elsworth	M and F	NA	European	62991	2018	9
ukb‐b‐16202	Carotene	Ben Elsworth	M and F	NA	European	64979	2018	16
ukb‐b‐7864	Vitamin B6	Ben Elsworth	M and F	NA	European	64979	2018	18
ukb‐b‐11349	Folate	Ben Elsworth	M and F	NA	European	64979	2018	15
ukb‐b‐19524	Vitamin B12	Ben Elsworth	M and F	NA	European	64979	2018	10
ukb‐b‐19390	Vitamin C	Ben Elsworth	M and F	NA	European	64979	2018	11
ebi‐a‐GCST90025967	Vitamin D	Cantin Baron	NA	34226706	European	418691	2021	152
ukb‐b‐6888	Vitamin E	Ben Elsworth	M and F	NA	European	64979	2018	12
ebi‐a‐GCST90025990	Calcium	Cantin Baron	NA	34226706	European	400792	2021	311
ukb‐b‐20447	Iron	Ben Elsworth	M and F	NA	European	64979	2018	14
ieu‐a‐1079	Zinc	David M. Evans	M and F	23720494	European	2603	2013	8
ieu‐a‐1077	Selenium	David M. Evans	M and F	23720494	European	2603	2013	6
ieu‐a‐1073	Copper	David M. Evans	M and F	23720494	European	2603	2013	6
ukb‐b‐7372	Magnesium	Ben Elsworth	M and F	NA	European	64979	2018	19
ukb‐b‐17881	Potassium	Ben Elsworth	M and F	NA	European	64979	2018	16
GCST90399568	Restless legs syndrome	Schormair B	M and F	38839884	European	27050	2022	12

### 2.3 Instrumental Variables

MR analysis relies on three core assumptions: relevance, independence, and exclusion restriction. In this study, we selected instrumental variables (IVs) from summary‐level GWAS data on serum micronutrients relevant to the pathophysiology of RLS. To satisfy the relevance assumption, we identified single nucleotide polymorphisms (SNPs) significantly associated with the micronutrient levels using a stringent significance threshold of *p* < 5 × 10^−6^. To meet the independence assumption, we performed linkage disequilibrium clumping with a window size of 10,000 kb and an *r* ^2^ cutoff of 0.001 to remove correlated SNPs. We assessed instrument strength by calculating the *F*‐statistic for each SNP, retaining those with *F* > 10, following the methodology described by Pierce and Burgess (2013). For the bidirectional analyses, the same filtering criteria were applied to the RLS GWAS to construct IVs when RLS served as the exposure. To address the exclusion restriction assumption, we conducted sensitivity analyses to test for horizontal pleiotropy, ensuring that causal inferences were not biased by pleiotropic effects. Detailed information on IVs is available in Table S1.

### 2.4 Statistical Analysis

We conducted MR analyses to investigate the causal links between serum micronutrient levels and RLS. Horizontal pleiotropy and outlier variants were detected and corrected with RadialMR and MR‐PRESSO. For the primary MR analysis, we used the inverse‐variance weighted (IVW) method under the assumption that all genetic instruments are valid and there is no horizontal pleiotropy. To enhance the robustness of our findings and address potential biases, we also applied complementary MR methods, including the weighted median method, MR‐Egger regression, IVW Radial, and RAPS, which are robust against weak instruments and pleiotropy. We applied a two‐tier framework that gives priority to FDR‐controlled IVW results while retaining the original requirement for directional concordance across complementary estimators: (1) Robust causal association. The IVW estimate meets the threshold FDR < 0.05 and the weighted median, MR Egger, radial IVW, and RAPS estimates point in the same direction of effect. (2) Suggestive causal association. The IVW estimate shows a raw *p* < 0.05 but FDR ≥ 0.05, with the effect direction supported by the weighted median, MR Egger, radial IVW, and RAPS. Associations not fulfilling either definition are regarded as providing no evidence of causality. To further assess directionality and isolate independent effects, we pre‐specified two secondary analyses for every micronutrient that attained at least a suggestive association. First, bidirectional MR was undertaken by reversing the exposure–outcome orientation—treating RLS as the exposure and the nutrient as the outcome; the absence of a reciprocal effect reinforced the plausibility of a nutrient‐to‐RLS causal pathway. Second, MVMR was applied whenever two or more nutrients met the suggestive threshold, entering those exposures simultaneously to generate conditionally independent effect estimates and to minimize bias arising from shared genetic architecture.

### 2.5 Sensitivity Analysis

To evaluate potential heterogeneity and pleiotropy in our results, we conducted several sensitivity analyses. Heterogeneity among the IVs (SNPs) was assessed using Cochran's *Q* test; a *p* value above 0.05 indicates no significant heterogeneity. Horizontal pleiotropy was examined using the MR‐Egger intercept test; a significant intercept (*p* value < 0.05) suggests the presence of directional pleiotropy.

Additionally, we applied the RadialMR approach, implementing both the IVW and MR‐Egger methods through the RadialMR package (version 1.1), to identify and exclude outlier SNPs that might bias the results due to pleiotropic effects. After removing these variance outliers, we re‐analyzed the data to correct for pleiotropy bias. To ensure the robustness of our findings, we performed a leave‐one‐out analysis by sequentially removing each SNP and re‐estimating the causal effect to assess whether any single SNP had a disproportionate influence on the results. For validation of the causal relationships, we confirmed that: (a) the MR‐Egger intercept did not indicate significant directional pleiotropy; (b) Cochran's *Q* test did not reveal significant heterogeneity.

### 2.6 Ethical Approval

This research utilized publicly available summary data from prior GWAS. The original studies had obtained ethical approvals and informed consent from their respective institutional review boards and participants. Since our research involved the analysis of existing, de‐identified data, no new ethical approval was required. All data were used following the relevant guidelines and regulations provided by the data sources.

## 3 Results

### 3.1 Causal Associations Between Micronutrients and RLS

We investigated the causal relationships between 15 serum micronutrients and RLS using MR analyses. Our rigorous SNP selection process identified between 6 and 311 SNPs for each micronutrient phenotype—specifically, 6 SNPs for retinol, 15 for folate, and 19 for magnesium. The F‐statistics for these IVs ranged from 18.08 to 3545, indicating that the instruments were sufficiently strong to minimize concerns about weak instrument bias (Table S1).

**FIGURE 2:**
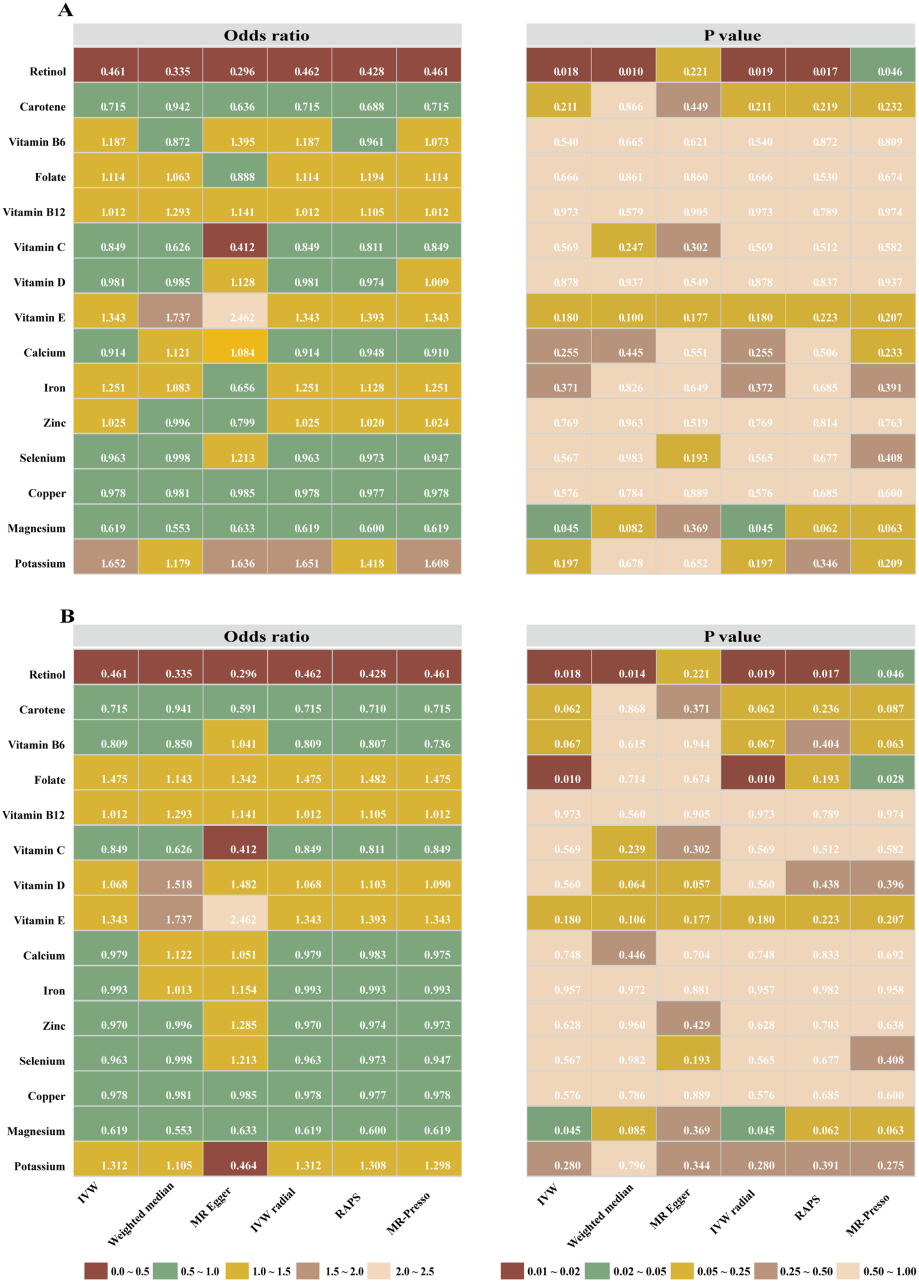
Heatmaps of MR analysis results evaluating the causal relationship between micronutrient levels and restless legs syndrome before and after outlier removal. Panel A shows the MR analysis results without outlier removal, whereas panel B presents the results after outliers were excluded using RadialMR analysis. Both panels include odds ratios (left) and corresponding *p* values (right), analyzed using six MR methods: Inverse Variance Weighted (IVW), Weighted Median, MR Egger, IVW Radial, Robust Adjusted Profile Score (RAPS), and MR‐Presso. The odds ratios and *p* values are color‐coded based on effect size.

**FIGURE 3:**
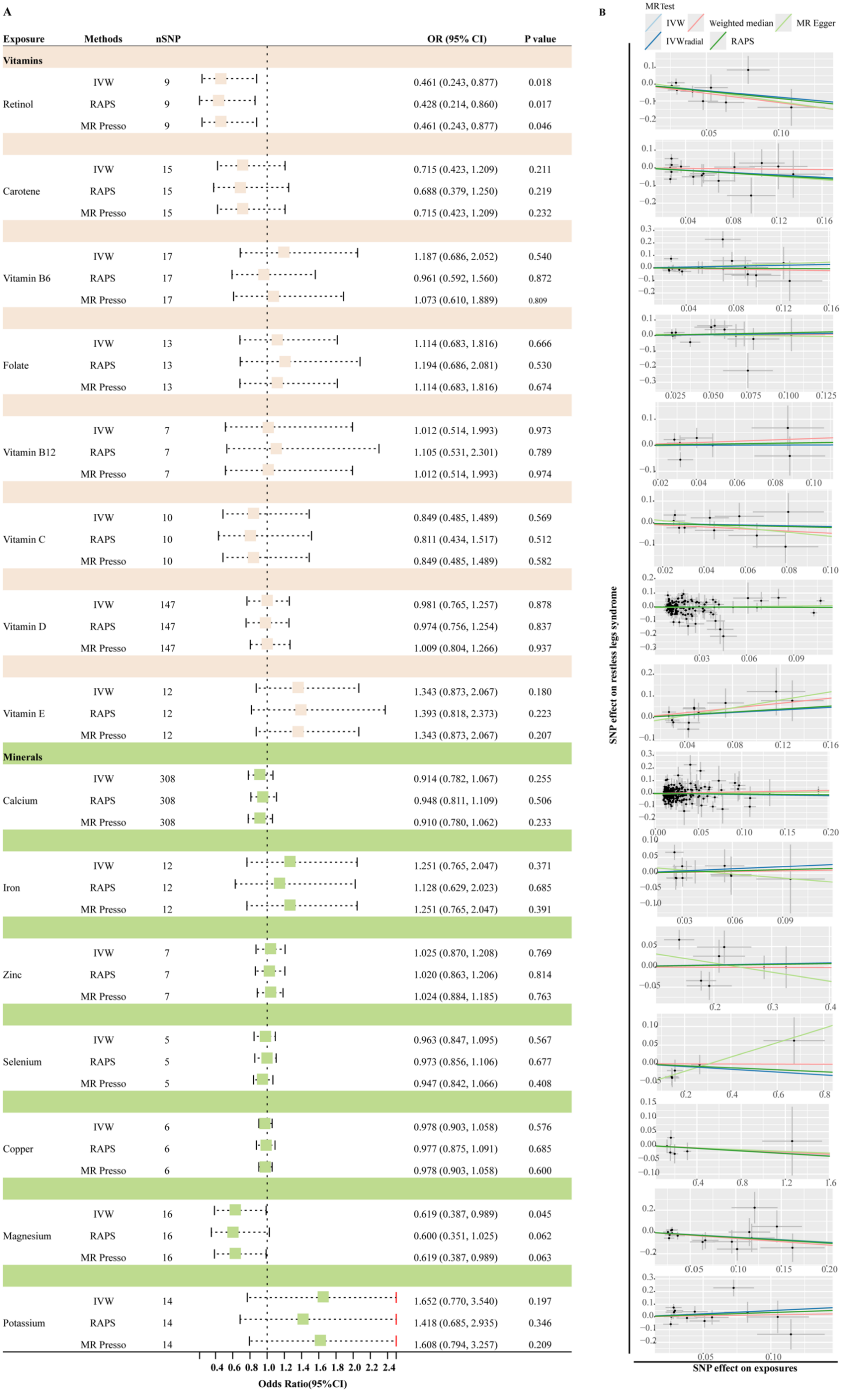
Forest plots and scatter plots of MR analysis results evaluating the causal relationship between micronutrient levels and restless legs syndrome. Panel A presents forest plots of odds ratios (ORs) with 95% confidence intervals (CIs) and corresponding *p* values for each micronutrient, assessed using three MR methods: inverse variance weighted (IVW), robust adjusted profile score (RAPS), and MR‐Presso. Panel B displays scatter plots illustrating horizontal pleiotropy, showing the SNP‐specific effects on exposures against their effects on RLS for each micronutrient. Lines in the scatter plots represent the causal estimates derived from IVW, MR Egger, weighted median, IVW radial, and RAPS methods.

**FIGURE 4:**
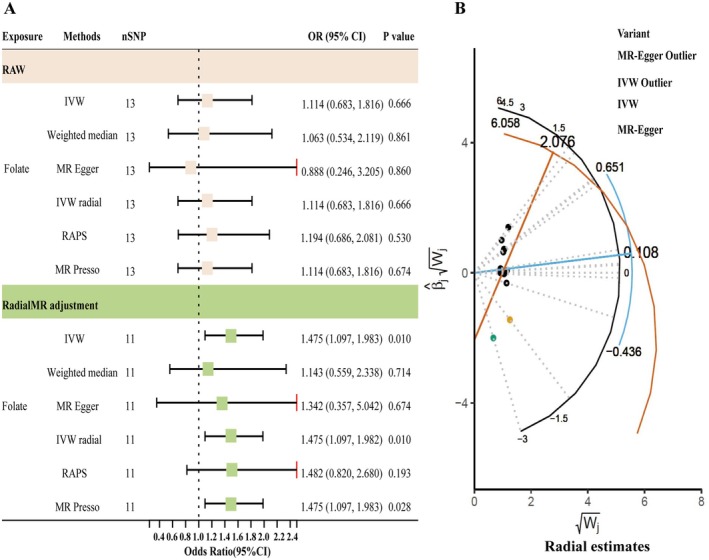
MR analysis results for folate and restless legs syndrome before and after outlier removal by RadialMR adjustment, and summary of causal associations for all micronutrients. (A) Shows forest plots of odds ratios with 95% confidence intervals and corresponding *p* values for folate and RLS, comparing results before and after RadialMR adjustment. (B) Displays the analysis of the radial estimates for folate, illustrating the identification and adjustment of outlier SNPs.

Under our two‐tier analytical framework, no nutrient met the robust criterion defined as an IVW *q*‐value below 0.05 (Table S2). After outlier removal, the smallest adjusted *q*‐values were observed for folate (*q* = 0.148), retinol (*q* = 0.274), and magnesium (*q* = 0.335); none met the 0.05 threshold. Three nutrients, however, met the criteria for the suggestive tier. Specifically, we observed a potential inverse association between serum levels of retinol and magnesium with the risk of RLS, implying that higher concentrations may confer a protective effect. For retinol, the IVW analysis yielded an odds ratio (OR) of 0.46 (95% CI: 0.25–0.86; raw *p* = 0.018; *q* = 0.274), with consistent directionality observed across the weighted‐median, MR‐Egger, IVW‐radial, and RAPS models (Figures 2A and 3A). Similarly, magnesium demonstrated an IVW OR of 0.62 (95% CI: 0.39–0.98; *p* = 0.045; *q* = 0.335), with supportive findings from complementary estimators. In contrast, folate exhibited a suggestive positive association with RLS risk. Upon removal of outlier SNPs via RadialMR (rs79748722, rs76630415; Table S3), the IVW estimate shifted towards a positive direction (OR = 1.48; *p* = 0.010; *q* = 0.148), corroborated by concordant directional effects in complementary models (Figure 4A,B). All remaining micronutrients demonstrated IVW *p* values ≥ 0.05 (Figure 2B; Table S4), with minimal changes post outlier removal. To evaluate potential reverse causality, we conducted reverse‐direction MR analyses using RLS as the exposure and each of the three nutrients with suggestive associations—retinol, magnesium, and folate—as outcomes. No evidence of reverse association was observed for any of these nutrients (all IVW raw *p* values > 0.2793), thereby reinforcing the inferred directionality from nutrient levels to RLS risk (Table S5). Given their suggestive associations with RLS, retinol, magnesium, and folate were further analyzed using an MVMR framework. Conditional *F*‐statistics ranged from 1.22 to 5.32, suggesting possible weak‐instrument bias and warranting cautious interpretation (Table 2). No evidence of directional pleiotropy was found (MR‐Egger intercept *p* = 0.750). After mutual adjustment, folate remained positively associated with RLS (OR = 2.88; 95% CI: 1.07–7.77; *p* = 0.037), magnesium showed a continued inverse association (OR = 0.38; 95% CI: 0.15–0.98; *p* = 0.045), whereas the effect of retinol was attenuated (OR = 0.55; 95% CI: 0.30–1.01; *p* = 0.055).

**TABLE 2:** Multivariable Mendelian randomization estimates for folate, retinol, and magnesium to restless legs syndrome.

Exposure	SNPs (*n*)	OR (95% CI)	*p*	*F*‐statistics
Folate	10	2.88 (1.07–7.77)	0.0368	1.71
Retinol	9	0.55 (0.30–1.01)	0.0547	5.32
Magnesium	16	0.38 (0.15–0.98)	0.0452	1.22

**FIGURE 5:**
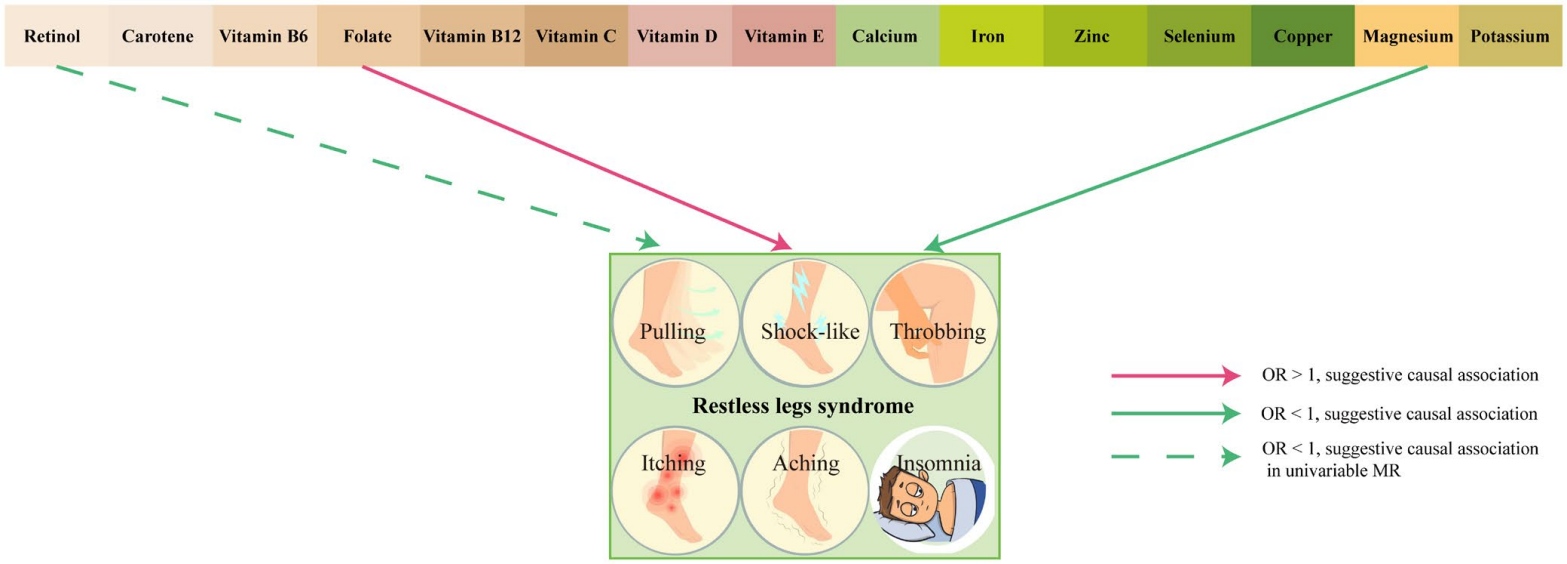
Overview of suggestive Mendelian randomization signals linking circulating micronutrients to restless legs syndrome (RLS). Colored arrows indicate micronutrients with suggestive causal evidence (IVW raw *p* < 0.05, FDR ≥ 0.05). A green solid line corresponds to an inverse association (OR < 1) that persists after multivariable MR (magnesium). A red solid line indicates a positive association (OR > 1) that remains significant after multivariable MR (folate). A green dashed line represents an inverse association (OR < 1) observed only in univariable MR (retinol). Micronutrients without arrows showed no evidence of a causal relationship (IVW *p* ≥ 0.05). FDR, Benjamini–Hochberg false discovery rate.

The overall causal relationships between micronutrients and RLS, as summarized in Figure 5, indicate that magnesium and folate exhibit suggestive causal associations—with inverse and positive directions, respectively—while the association between retinol and RLS was attenuated to borderline significance following multivariable adjustment.

### 3.2 Sensitive Analysis

To assess the robustness of our findings and identify potential heterogeneity and horizontal pleiotropy, we conducted several sensitivity analyses. Heterogeneity among the IVs (SNPs) was evaluated using Cochran's *Q* test within the IVW model. Before the removal of outlier SNPs identified by RadialMR, *p* values ranged from 0.001 to 0.733. Notably, Vitamin D, calcium, zinc, and potassium exhibited significant heterogeneity, with *p* values between 0.001 and 0.040. After excluding outlier SNPs using RadialMR, no significant heterogeneity was detected, as *p* values ranged from 0.239 to 1.000. These results are summarized in Table 3, with detailed information provided in Tables S6 and S7.

**TABLE 3:** Sensitivity analysis results of micronutrient levels and restless legs syndrome.

Exposures	SNPs (*n*)	Directional pleiotropy (raw)		Cochran's *Q* test (raw)		SNPs (*n*)	Directional pleiotropy (RadialMR adjustment)		Cochran's *Q* test (RadialMR adjustment)	
		Egger intercept	*p*	*Q* statistic	*p*		Egger intercept	*p*	*Q* statistic	*p*
Retinol	9	0.018	0.613	8.032	0.430	9	0.018	0.613	8.032	0.430
Carotene	15	0.006	0.821	15.658	0.335	13	0.010	0.707	5.151	0.953
Vitamin B6	17	−0.007	0.789	23.303	0.106	15	−0.011	0.626	3.086	0.999
Folate	13	0.009	0.712	10.464	0.575	11	0.004	0.880	2.734	0.987
Vitamin B12	7	−0.005	0.907	6.031	0.420	7	−0.005	0.907	6.031	0.420
Vitamin C	10	0.028	0.362	8.554	0.479	10	0.028	0.362	8.554	0.479
Vitamin D	147	−0.004	0.369	206.589	0.001	132	−0.009	0.050	114.123	0.853
Vitamin E	12	−0.027	0.309	8.137	0.701	12	−0.027	0.309	8.137	0.701
Calcium	308	−0.005	0.121	366.287	0.011	281	−0.002	0.505	203.912	1.000
Iron	12	0.023	0.466	8.688	0.651	11	−0.005	0.868	1.870	0.997
Zinc	7	0.054	0.461	13.174	0.040	6	−0.066	0.370	5.404	0.369
Selenium	5	−0.059	0.107	5.510	0.239	5	−0.059	0.107	5.510	0.239
Copper	6	−0.002	0.929	2.785	0.733	6	−0.002	0.929	2.785	0.733
Magnesium	16	−0.001	0.957	14.069	0.520	16	−0.001	0.957	14.069	0.520
Potassium	14	0.000	0.992	29.722	0.005	11	0.042	0.178	7.334	0.694

Horizontal pleiotropy was assessed using the MR‐Egger intercept test. The test indicated no evidence of directional pleiotropy, with *p* values between 0.106 and 0.992 before outlier removal, and between 0.050 and 0.957 following RadialMR correction. The summarized results are presented in Table 3, with additional details in Tables S8 and S9.

**FIGURE 6:**
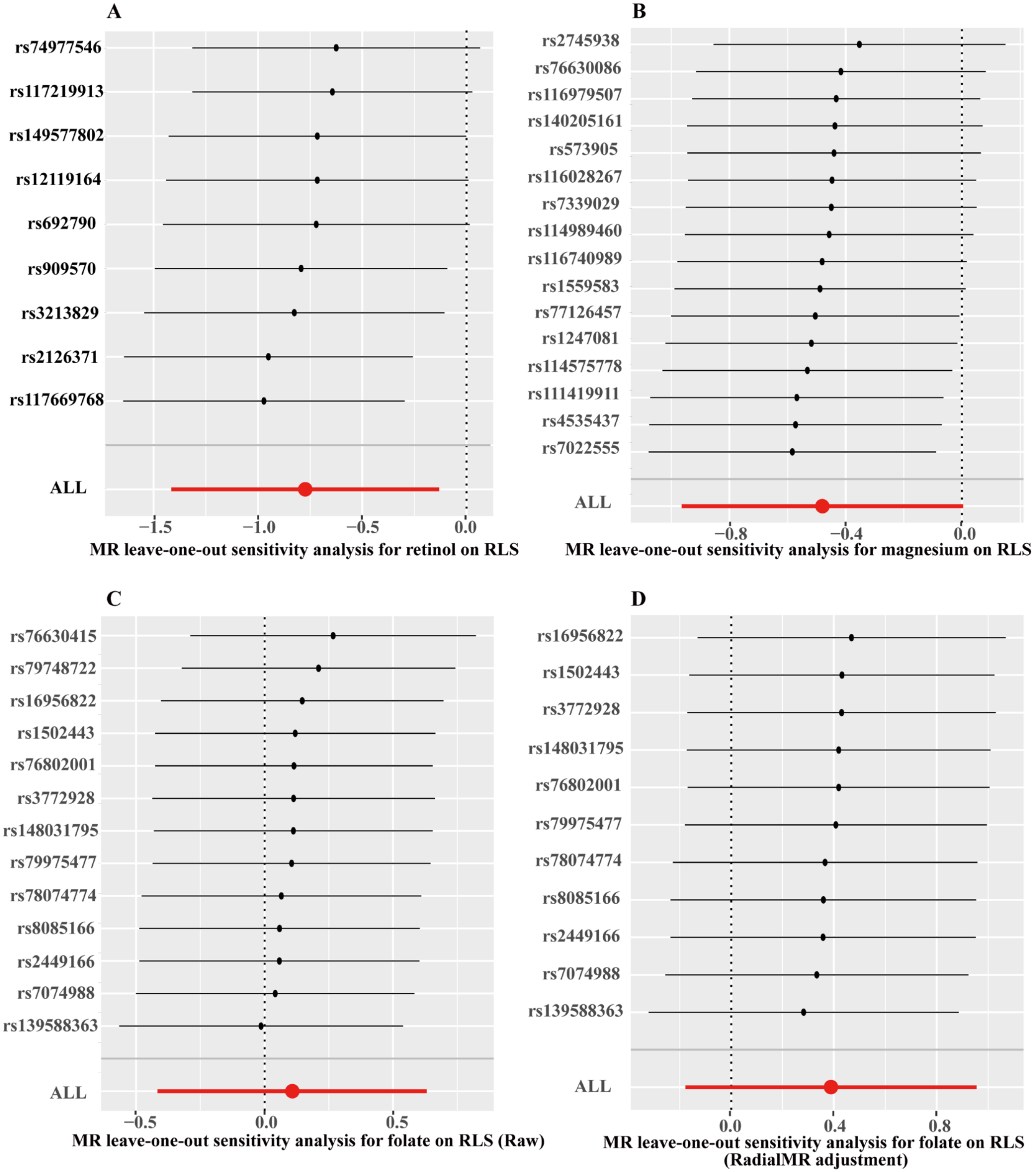
Leave‐one‐out sensitivity analysis for retinol, magnesium, and folate with restless legs syndrome. Panel (A, B) present the leave‐one‐out sensitivity analysis results for retinol and magnesium, respectively. (C) The analysis for folate before RadialMR adjustment (Raw), whereas (D) presents the results after RadialMR adjustment. Each point represents the causal effect estimate after removing a specific SNP, with the red dot (“ALL”) indicating the overall causal effect including all SNPs. These analyses assess the robustness of the MR results and identify potential influential SNPs driving the observed associations.

Scatter plots depicting the regression slopes of individual SNPs for each micronutrient‐RLS association are shown in Figure 3B. Furthermore, we performed a leave‐one‐out analysis to determine whether any single SNP disproportionately influenced the overall causal estimates for retinol, folate, and magnesium on RLS. The analysis confirmed that the associations were not driven by any individual SNP (Figure 6); supporting the stability of our results.

## 4 Discussion

In this research, we utilized an integrated two‐sample, bidirectional, and multivariable MR framework to probe the causal contribution of 15 circulating micronutrients to RLS. Forward MR identified no “robust” signals after false‐discovery control, but three nutrients reached our suggestive tier: genetically higher retinol and magnesium were associated with lower RLS risk, whereas higher folate was associated with higher risk once pleiotropic outliers had been removed. Reverse MR analysis provided no evidence of reverse causality. In the multivariable MR model, folate remained positively associated with RLS risk, whereas magnesium retained an inverse association, whereas the effect of retinol attenuated to borderline significance. Collectively, these findings implicate folate excess and magnesium insufficiency as independent, potentially modifiable contributors to RLS pathophysiology and suggest that retinol warrants further study.

### 4.1 Magnesium and RLS Protection

Our analysis indicated a suggestive negative causal association between magnesium levels and RLS, suggesting that higher magnesium levels may confer a protective effect against the disorder. Clinical studies have investigated magnesium supplementation as a potential treatment for RLS, yielding mixed results. An open‐label pilot study reported that magnesium citrate supplementation alleviated RLS symptoms and improved quality of life measures, highlighting its potential therapeutic role (Gorantla et al. 2024). Similarly, a randomized controlled trial found that magnesium supplementation significantly reduced RLS symptoms and enhanced sleep quality (Jadidi et al. 2022). Conversely, some studies have not observed significant improvements with magnesium supplementation; however, benefits in specific subgroups cannot be entirely ruled out (Marshall et al. 2019). These inconsistent findings imply that while magnesium may play a role in RLS management, its efficacy may vary based on individual patient characteristics and baseline magnesium levels.

To understand these clinical observations, it is important to consider the biological functions of magnesium. Magnesium is essential for neuromuscular function, acting as a natural calcium antagonist to facilitate muscle relaxation and prevent excessive neuronal excitability (Mathew and Panonnummal 2021; Souza et al. 2023). A magnesium deficiency has been linked to increased muscle cramps and RLS symptoms, aligning with our findings (Stanojevic et al. 2024). Additionally, magnesium modulates neurotransmitter release and helps maintain the balance of excitatory and inhibitory signals in the central nervous system, which may underpin its protective effects in RLS (Mathew and Panonnummal 2023; Sears and Hewett 2021). Its involvement in regulating circadian rhythms could also influence the timing and severity of RLS symptoms, which typically worsen during rest or inactivity (Feeney et al. 2016).

To confirm magnesium's protective role in RLS, additional studies with larger sample sizes and diverse populations are necessary. Interventional trials examining magnesium supplementation could provide additional insights into its potential as a therapeutic agent for RLS management.

### 4.2 Folate and RLS Association

Our analysis revealed a suggestive positive causal association between genetically predicted folate levels and RLS risk. This finding is notable, and initially appears to contrast with certain clinical observations, prompting a deeper examination of the multifaceted relationship between folate and RLS. A recent 2025 meta‐analysis found no difference in circulating folate between idiopathic RLS cases and controls, and no contemporary trial shows symptomatic benefit from folic acid supplementation outside frank deficiency (Xu et al. 2025). In contrast, low folate contributes to pregnancy‐related RLS, where supplementation (with iron) lowers risk (Miyazaki et al. 2023). Thus, in non‐pregnant adults with normal folate status, additional folic acid rarely improves RLS, whereas correcting deficiency in pregnancy or malnutrition remains important.

Our MR study offers a different lens through which to view this association. By utilizing genetic variants as proxies for lifelong folate level exposure, MR analysis minimizes confounding inherent in observational studies and is less susceptible to reverse causation (where RLS might influence folate levels or intake). Therefore, our finding of a positive causal association between genetically higher folate levels and RLS risk does not necessarily contradict the observed benefits of folate supplementation in deficient individuals. Instead, it may point toward different biological consequences depending on the context: correcting a deficiency versus a lifelong tendency toward higher systemic folate levels. To reconcile these perspectives, it is essential to explore the underlying biological mechanisms. Folate is crucial for DNA synthesis, repair, and methylation, and its dysregulation has been implicated in various neurological disorders (Irwin et al. 2016; Lintas 2019). The positive association observed in our MR study might reflect a complex interplay where chronically elevated folate levels, as genetically predisposed, could influence neurological function differently than acute supplementation. For instance, persistently higher folate levels could subtly alter neurotransmitter synthesis pathways (e.g., those involving dopamine, which is implicated in RLS) or neuronal excitability over extended periods (Balashova et al. 2018; Liwinski and Lang 2023). It is also conceivable that very high folate levels, potentially driven by genetic predisposition and amplified by high dietary intake or supplementation, could disrupt the delicate balance of other B vitamins, such as vitamin B12, which is crucial for neurological health, thereby indirectly contributing to RLS risk (Fardous and Heydari 2023; Miller et al. 2024). This is distinct from the therapeutic effect of normalizing folate levels in a deficient state.

Future work should test these hypotheses in longitudinal cohorts that combine genetic scores with measured folate intake, explore non‐linear associations, and examine interactions with B12 and iron. Such studies will clarify whether personalized folate recommendations are warranted for individuals who are genetically predisposed to higher folate levels and, potentially, to RLS.

### 4.3 Retinol and RLS Protection

Our initial univariable MR study identified a suggestive negative causal association between retinol levels and RLS. However, in subsequent MVMR analysis, which adjusted for the effects of genetically predicted folate and magnesium levels, an association between retinol and RLS was attenuated (*p* = 0.055). This finding appears to be novel, as current literature does not extensively explore the relationship between retinol and RLS.

Retinol is well‐documented for its neuroprotective and anti‐inflammatory properties (Das et al. 2019; Mohammadzadeh Honarvar et al. 2017), which provided the rationale for investigating its potential role in RLS. Retinol is essential for neuronal health and function, influencing the expression of genes related to dopamine regulation, such as the tyrosine hydroxylase (TH) gene, and synaptic plasticity, including genes like brain‐derived neurotrophic factor (BDNF) (Katsuki et al. 2009; Kunzler et al. 2019; Lim et al. 2020). Additionally, its antioxidant capabilities may help mitigate oxidative stress, a factor implicated in various neurological disorders, including RLS (Elomda et al. 2018).

Although these biological functions highlight retinol's importance for neurological health, the results from our MVMR analysis suggest that an independent protective effect of circulating retinol levels on RLS risk may be limited or less pronounced when considered alongside the stronger influences of folate and magnesium. The initial suggestive association observed in the univariable setting might have been partly confounded by these other micronutrients.

Direct studies comprehensively evaluating retinol's role in RLS are scarce. Although our MVMR analysis, after adjusting for folate and magnesium, did not confirm a statistically significant independent protective effect of retinol, its fundamental roles in neuronal maintenance and inflammation reduction (as previously described) suggest that further clarification of its potential relevance to RLS may still be warranted. Future research could focus on elucidating any subtle or context‐dependent biological mechanisms of retinol with RLS. This could include exploring potential complex interactions between retinol and other micronutrients (such as folate and magnesium), or investigating whether retinol status is particularly relevant for specific RLS subpopulations or under particular deficiency conditions. However, as our adjusted analysis indicates, interventions focused solely on elevating retinol levels for RLS prevention do not appear to be a primary strategy based on the current evidence of its independent contribution.

### 4.4 Other Micronutrients and RLS: Discrepancies and Future Directions

Iron has long been recognized as a critical factor in the pathophysiology of RLS. A substantial body of evidence, including recent reviews, indicates that iron deficiency, particularly within the brain rather than systemically, is strongly associated with the development and severity of RLS symptoms (Alzaabi et al. 2025; Connor et al. 2017; Silber et al. 2021). Iron is essential for dopamine synthesis and metabolism; consequently, its deficiency can lead to dopaminergic dysfunction, a key neuropathological feature implicated in RLS (Alzaabi et al. 2025; Bugnicourt 2024; González‐Parejo et al. 2024). Despite iron's established pivotal role in RLS, our MR investigation found no suggestive causal link between genetically predicted serum iron levels and RLS. This discrepancy likely arises because serum iron is an imperfect proxy for central nervous system (CNS) iron status, the latter being more critical to RLS pathophysiology (Alzaabi et al. 2025; Silber et al. 2021). The genetic instruments for serum iron used in our study might primarily reflect systemic iron regulation and not adequately capture variations in brain iron. This could mask a true association if brain iron is the key mediator. Therefore, future MR studies should prioritize instruments or direct measures more specific to CNS iron status to better evaluate the iron hypothesis in RLS.

Beyond iron, observational studies on other micronutrients in RLS show varied findings. For example, some observational studies have reported higher serum calcium, copper, and magnesium levels in RLS patients (Jimenez‐Jimenez et al. 2022). Evidence for zinc is conflicting: while some research has noted elevated levels, other studies found no significant difference in serum zinc (Chen et al. 2021). Despite such observational reports for certain micronutrients, our MR analysis found no causal effects of genetically predicted serum levels of zinc, calcium, copper, potassium, or other tested nutrients on RLS risk. These null MR findings for other micronutrients suggest that their routine supplementation is unlikely to prevent idiopathic RLS in the general population. Nevertheless, these results do not preclude the possibility of clinically important effects in specific contexts, such as pronounced deficiency states or particular patient subgroups where nutrient handling might differ (Jimenez‐Jimenez et al. 2022). Furthermore, potential roles mediated through brain‐specific regulatory mechanisms, which may not be well represented by genetic instruments for blood‐based nutrient levels, cannot be ruled out. Future work should therefore aim to incorporate CNS‐focused genomic measures (e.g., expression QTLs for nutrient transporters), expand GWAS sample sizes for lesser‐studied analytes, and explore gene–environment interactions that might reveal context‐dependent micronutrient effects on RLS.

### 4.5 Limitations

Our study has several limitations. First, findings are primarily based on individuals of European ancestry, limiting generalizability. Second, while we conducted extensive sensitivity analyses, MR relies on assumptions that may not fully hold in practice. Third, serum micronutrient levels may not perfectly reflect tissue‐specific or CNS concentrations, which are critical for RLS. This was particularly evident for iron, where brain‐specific status is more relevant. Additionally, our multivariable MR analysis showed potential weak‐instrument bias, suggesting caution in interpreting those specific results. Finally, MR identifies statistical associations but does not reveal underlying biological mechanisms, requiring further experimental research.

## 5 Conclusion

In summary, our MR study suggests that genetically predicted higher magnesium levels may be protective against RLS, whereas higher folate levels show a positive association. The initial protective link for retinol weakened after multivariable adjustment, suggesting a less independent role. These findings highlight the potential contribution of specific micronutrients to RLS and hint at complex nutritional avenues for intervention. Although acknowledging limitations, our work underscores the value of genetic approaches. Future research should validate these links in diverse groups, explore mechanisms, and assess clinical efficacy.

## Author Contributions

**Yun Lin:** conceptualization (equal), data curation (equal), investigation (equal), methodology (equal), visualization (equal), writing – original draft (lead). **Xiaorui Cai:** investigation (equal), validation (equal), writing – original draft (supporting). **Xiaoling Tang:** conceptualization (equal), investigation (equal), methodology (equal), project administration (equal), writing – review and editing (equal). **Haohao Chen:** conceptualization (equal), data curation (equal), investigation (equal), methodology (equal), visualization (equal), writing – review and editing (equal).

## Conflicts of Interest

The authors declare no conflicts of interest.

## Supporting information


**Table S1:** fsn370568‐sup‐0001‐TableS1.xlsx.

**Table S2:** fsn370568‐sup‐0002‐TableS2.xlsx.

**Table S3:** fsn370568‐sup‐0003‐TableS3.xlsx.

**Table S4:** fsn370568‐sup‐0004‐TableS4.xlsx.

**Table S5:** fsn370568‐sup‐0005‐TableS5.xlsx.

**Table S6:** fsn370568‐sup‐0006‐TableS6.xlsx.

**Table S7:** fsn370568‐sup‐0007‐TableS7.xlsx.

**Table S8:** fsn370568‐sup‐0008‐TableS8.xlsx.

**Table S9:** fsn370568‐sup‐0009‐TableS9.xlsx.

## Data Availability

The data that support the findings of this study are publicly available. Genetic summary statistics for the 15 analyzed micronutrients were obtained from the IEU Open GWAS Project database, accessible at https://gwas.mrcieu.ac.uk/. The specific GWAS identifiers for each micronutrient are listed in Table 1 of the manuscript. Summary statistics for Restless Legs Syndrome (RLS) were acquired from the GWAS Catalog under accession number GCST90399568, accessible at https://www.ebi.ac.uk/gwas/studies/GCST90399568. The original studies that generated these datasets received ethical approval and informed consent from their respective institutional review boards and participants. The current research involved the analysis of existing, de‐identified summary‐level data; therefore, no new ethical approval was required. All data were utilized in accordance with the relevant guidelines and regulations of the data sources.
